# The neuroprotective effect of picroside II via regulating the expression of myelin basic protein after cerebral ischemia injury in rats

**DOI:** 10.1186/1471-2202-15-25

**Published:** 2014-02-14

**Authors:** Li Zhao, Yunliang Guo, Xiaojun Ji, Meizeng Zhang

**Affiliations:** 1 Institute of Cerebrovascular Diseases, Affiliated Hospital of Qingdao University, Qingdao, Shandong 266003, China

**Keywords:** Picroside II, Therapeutic dose, Time window, Cerebral ischemia, MBP, Rats

## Abstract

**Background:**

To explore the neuroprotective effect and optimize the therapeutic dose and time window of picroside II by orthogonal test and the expression of myelin basic protein (MBP) in cerebral ischemic injury in rats. Bilateral common carotid artery occlusion (BCCAO) was used to establish forebrain ischemia models. The successful rat models were grouped according to orthogonal experimental design and injected picroside II intraperitoneally at different ischemic time with different doses. Myelin sheath fast green staining(FGS) and transmission electron microscopy (TEM) were used to observe nerve fiber myelin; the expression of MBP was tested qualitatively and quantitatively by immunohistochemical assay (IHC) and Western blot (WB); Reverse transcription polymerase chain reaction (RT-PCR) was used to detect the transcription level of MBP mRNA.

**Results:**

The protective effect of picroside II was presented by increasing the expression of MBP and decreasing demyelination after cerebral ischemic injury. The best therapeutic time window and dose was (1) ischemia 2.0 h with picroside II 10 mg/kg body weight according to the results of FGS, IHC and WB; (2) ischemia 1.5 h with picroside II 20 mg/kg according to the analysis of RT-PCR.

**Conclusion:**

Given the principle of the longest time window and the lowest therapeutic dose, the optimized therapeutic dose and time window should be injecting picroside II intraperitoneally with 10-20 mg/kg body weight at ischemia 1.5-2.0 h in cerebral ischemic injury.

## Background

As an important myelin sheath structural protein in central nervous system (CNS) [1], myelin basic protein (MBP) lies in the serous surface of myelin sheath and closely integrates with the lipids of myelin and beneficial to steady the structure and function of myelin in CNS [2]. MBP has the specificity of nervous tissues [3], MBP lossing will lead to myelination obstacles and its level can reflect the severity of the damage of CNS and myelin [4], so that sufficient MBP is important for the function recovery of the CNS [5]. Animal experiments [6] proved that there was a small amount expression of MBP mRNA in the brain of normal adult rats, while the MBP mRNA [7] and protein [8] decreased time-dependently in early stage of cerebral ischemic injury [9]. However, the expression of MBP mRNA will rise slightly as the ischemic time extension, and its expressing level at ischemia 7d was significantly higher than that at ischemia 1d. Previous study reported that acupuncture treatment could increase MBP expression and promote the regeneration of myelin [10]. Picroside II, an active ingredient of traditional Chinese medicine, has many neuroprotective effects of antioxidant, anti-inflammatory, anti-apoptosis [11,12], however, whether it could influence the expression levels of MBP or not has not been reported so far. Recently, we explored the treatment dose and time window of picroside II after cerebral ischemia reperfusion injury via the neurobehavioral function of rats, cerebral infarction volume and immunohistochemical staining and proved that injecting picroside II 20 mg/kg body weight intraperitoneally at ischemia 1.5 h could achieved a ideal therapeutic outcome for cerebral ischemic injury in rats [13,14]. In consideration of the limitation of neurobehavioral evaluation and immunohistochemical assay, we attempted to detect the expression levels of MBP in brain tissue qualitatively and quantitatively and observe the change of myelin structure through a various biological techniques, just to explore the optimal therapeutic dose and time window of picroside II after cerebral ischemic injury.

## Results

### Test results

**Figure 1:**
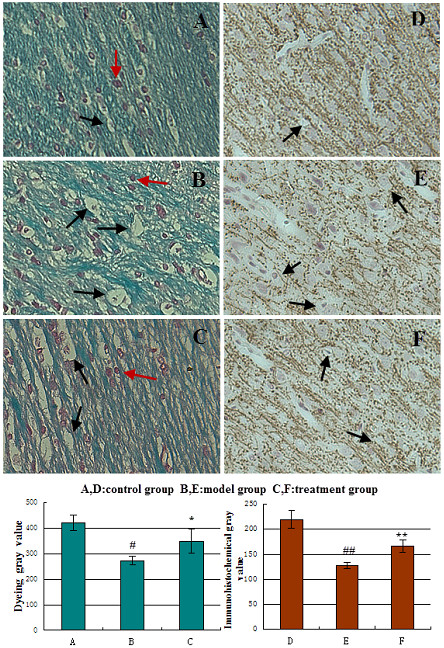
**Myelin nerve fibers in parietal white matter of rats, FGS × 400.** Normal myelin **(A)** showed cord-like, dark green, tightly packed, and cells was red (Red arrow); it showed myelin fibers loose and light-stained, glial cell vacuolation (Black arrow) after modeling **(B)**. In model group, myelin gray value (MGV) decreased significantly than that in control group (^#^*P* < 0.05 *vs* control group) and increased significantly than that in model group after treatment **(C)** (*P < 0.05 *vs* model group). The expression of MBP in parietal white matter, SABC × 400. In the control group **(D)**, myelin fibers was arranged closely and tidy. After modeling **(E)**, myelin degeneration release and disordered, positive cells showed cytoplasmic uneven coloring, and vesicular (Black arrow). The MBP expressed significantly lower in model group (^##^*P* < 0.05 *vs* control group) and increased significantly and myelin fibers tightly packed in treatment group **(F)** (**P < 0.05 *vs* model group).

**Figure 2:**
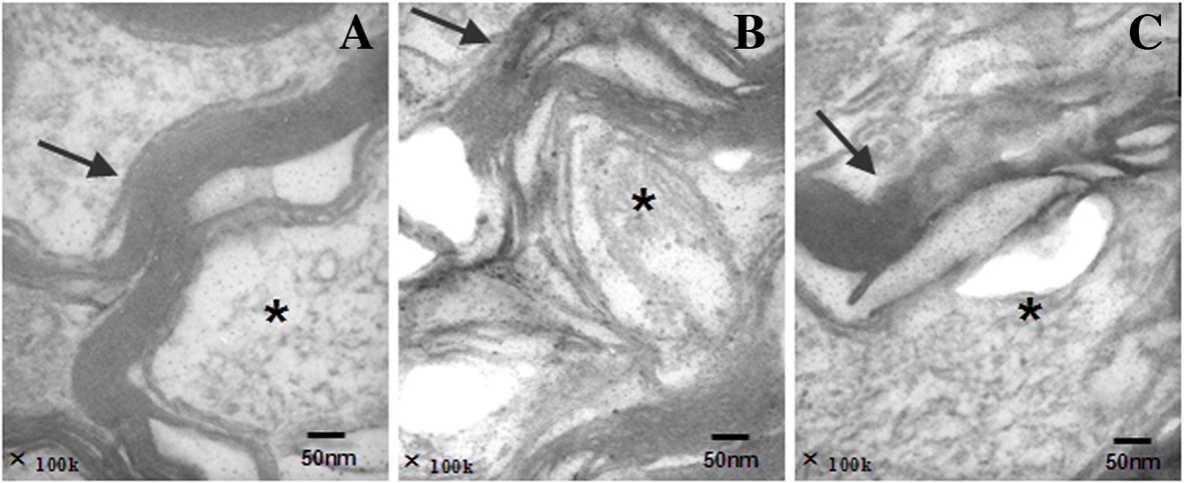
**Ultrastructure of myelin nerve fibers in ischemic cortical area of rats, TEM.** C: control group; M: model group; T: treatment group. The myelin sheath (Black arrow) and the neural axon (*) in control group were distinct and neat **(A)**, and unclear or disappeared in model group **(B)**, and those injuries was alleviated in treatment **(C)**.

**Figure 3:**
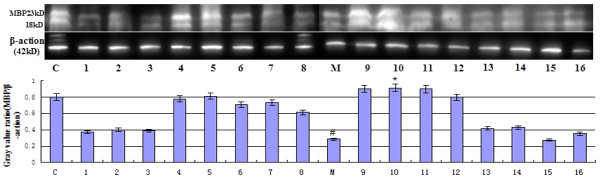
**The effect of picroside II on the expression of MBP detected by Western blot.** β-action was used as a loading paraqmeter. Line 1–16 were the treatment group rats which were treated in different time with different doses. In model group (M), the RCP of MBP was observably lower than that in control group (C) (^#^*P* < 0.05), while markedly increased after treatment by picroside II (*P < 0.05 *vs* model group).

**Figure 4:**
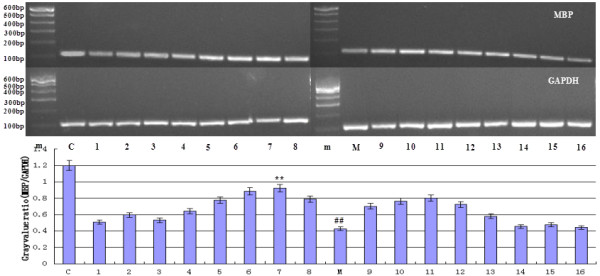
**The effect of picroside II on the transcription of MBP mRNA detected by RT-PCR.** GAPDH was used as a loading parameter. “m” presented marker. Line 1–16 were the treatment group rats which were treated in different time with different doses. In model group (M), the RAM of MBP mRNA was significantly decreased than that in control group (C) (^##^*P* < 0.05), while it was markedly increased after treatment (**P < 0.05 *vs* model group).

In control group, myelin showed cord-like, dark green, closely arranged after fast green staining. After modeling, myelin showed loose and light-stained, glial cell vacuolated, and myelin gray value (MGV), gray value of myelin (GVM), relative content of protein (RCP), relative abundance of mRNA (RAM) were lower markedly than those in control group (*t* = 13.79-26.13, *P* < 0.05). While MGV, GVM, RCP and RAM were significantly higher than those in model group after treated by picroside II (*t* = 3.09-3.71, *P* < 0.05)( Table 1, Figures 1, 2, 3 and 4). The orthogonal test results were shown in Table 2. Data listed in the Table 2 are the mean of three times of orthogonal experiment. Data of I, II, II, IV and SS were the ANOVA results of MGV, and the ANOVA results of rest indexes were omitted.

**Table 1:** The results of MGV, GVM, RCP and RAM (mean ± SD)

**Groups**	**n**	**MGV**	**GVM**	**RCP**	**RAM**
Control	5	421.674 ± 30.782	219.591 ± 17.693	0.801 ± 0.074	1.201 ± 0.099
Model	5	273.633 ± 17.421^#^	127.102 ± 6.350^#^	0.286 ± 0.026^#^	0.427 ± 0.057^#^
Treatment	16 × 3	349.043 ± 46.820*	166.263 ± 12.969*	0.612 ± 0.043*	0.662 ± 0.154*

^#^*P* < 0.05 *vs* control group, *P < 0.05 vs model group.

**Table 2:** **[L**_**16 **_**(4**^**5**^**)] orthogonal table and test results**

**Test no.**	**Rank no.**					**MGV**	**GVM**	**RCP**	**RAM**
	**A**	**B**	**C**	**D**	**E**				
1	1	1	1	1	1	283.654	127.002	0.373	0.507
2	1	2	2	2	2	297.874	136.098	0.402	0.596
3	1	3	3	3	3	290.544	137.056	0.39	0.531
4	1	4	4	4	4	320.887	146.417	0.777	0.643
5	2	1	2	3	4	342.117	158.028	0.812	0.777
6	2	2	1	4	3	389.432	190.337	0.707	0.885
7	2	3	4	1	2	396.745	184.114	0.734	0.923
8	2	4	3	2	1	379.667	178.013	0.612	0.789
9	3	1	3	4	2	388.798	189.100	0.901	0.702
10	3	2	4	3	1	420.765	207.135	0.912	0.765
11	3	3	1	2	4	400.456	202.007	0.897	0.801
12	3	4	2	1	3	398.567	195.243	0.798	0.723
13	4	1	4	2	3	330.734	161.477	0.417	0.579
14	4	2	3	1	4	341.100	169.549	0.43	0.455
15	4	3	2	4	1	310.967	147.608	0.276	0.476
16	4	4	1	3	2	292.374	131.03	0.352	0.444
SUM						5584.681	2660.214	9.790	10.596
I	1192.959	1345.303	1365.916	1420.066	1395.053				
II	1507.961	1449.171	1349.525	1408.731	1375.791				
III	1608.586	1398.712	1400.109	1345.800	1409.277				
IV	1275.175	1391.495	1469.131	1410.084	1404.560				
SS	28388.071	1356.219	2107.471	864.883	164.684				

### Results analysis

Myelin gray value (MGV) (Figure 1): The effects of different levels of variable factor A (time) on the injury of myelin showed a significant difference (*P* < 0.01), while no significant influence found among factor B (dose) and factor C (time-dose interaction) (*P* > 0.05). That is to say the administration time had significant impact on the degree of myelin damage after cerebral ischemia, but the influence of administration dose and time-dose interaction was negligible (Table 3). The pairwise comparisons of data at different levels according to LSD showed that the administration time between the groups were statistically significant (*P* < 0.05). The administration dose between 5 mg(B1) and 10 mg(B2) was statistically significant (*P* < 0.05), while the rest groups were of no statistical significance (*P* > 0.05). From the principle of lowest therapeutic dose with longest time window, the optimized composition were A3B2, that is to say the best therapeutic dose and time window was ischemia 2.0 h with picroside II 10 mg/kg body weight by intraperitoneally injection.

**Table 3:** ANOVA of MGV

**Source of variation**	**SS**	**Df**	**MS**	***F***	***P***
Time window	28388.071	3	9462.690	55.15	0.01
Drug dose	1356.219	3	452.073	2.64	0.14
Time × dose	2107.471	3	702.490	4.09	0.07
Error	1029.568	6	171.595		

### Gray value of myelin (GVM)

(Figure 1): There was a significant difference between the different levels of impact factors A (time window or ischemic time) on the expression of myelin (*P* < 0.05), while no significant probability (*P* > 0.05) found among impact factor B (drug dose) and factor C (time-dose interaction). This indicated the therapeutic time window (or ischemic time) had significantly influence on the expression of myelin after cerebral ischemia injury, while no significant influence existed in different drug doses and time-dose interactions (Table 4). LSD showed that different administration time between 1.0 h(A1) and 1.5 h(A2), 1.0 h(A1) and 2.0 h(A3), 1.5 h(A2) and 2.0 h(A3), 1.5 h(A2) and 2.5 h(A4), 2.0 h(A3) and 2.5 h(A4) had significant differences (*P* < 0.05), no significant differences found between the rest groups (*P* > 0.05). There was no statistical significance (*P* > 0.05) between groups on dose. So, the best combination is A3B2 (2.0 h/10 mg), that is injecting picroside II 10 mg/ kg body weight at cerebral ischemia 2.0 h.

**Table 4:** ANOVA of GVM

**Source of variation**	**SS**	**Df**	**MS**	***F***	***P***
Time window	8916.233	3	2972.078	28.77	0.01
Drug dose	638.716	3	212.905	2.06	0.21
Time × dose	560.222	3	186.741	1.81	0.25
Error	619.788	6	103.298		

Ultrastructure of myelin by TEM: In control group, the structure of myelin sheath was distinct and neat, and the neural axon was in focus; while unclear and irregular and the neural axon disappeared in model group; and the damaged myelin sheath in treatment group recovered significantly comparing with model group (Figure 2).

Relative content of protein (RCP) by WB: Quantitative detection of the expression of MBP showed that different intensity of MBP protein expressed in different groups (Figure 3), and it was significantly higher than that in model group after treatment. Analysis of variance showed that the different levels of factor A (time) had significant differences on the expression of MBP (*P* < 0.05), but no significant differences in factor B (dose) and factor C (time-dose interaction) (Table 5). LSD indicated that significant deviations (*P* < 0.05) were found between 1.0 h(A1) and 1.5 h(A2), 1.0 h(A1) and 2.0 h(A3), 1.5 h(A2) and 2.5 h(A4), 2.0 h(A3) and 2.5 h(A4) in therapeutic time window, but no significant deviations between the rest therapeutic time levels (*P* > 0.05). The different therapeutic dose levels had no significant differences (*P* > 0.05). Synthetically, the best combination is A3B2 (1.5 h/10 mg), i.e. the best therapeutic time window and dose of picroside II should be injecting intraperitoneally with 10 mg/kg body weight at cerebral ischemia 2.0 h.

**Table 5:** ANOVA of RCP

**Source of variation**	**SS**	**Df**	**MS**	***F***	***P***
Time window	0.625	3	0.208	12.84	0.01
Drug dose	0.009	3	0.003	0.18	0.91
Time × dose	0.052	3	0.017	1.06	0.43
Error	0.097	6	0.016		

Relative abundance of mRNA (RAM) by RT-PCR: The expressions of MBP mRNA differed between various groups, and increased markedly than that in model group after treatment (Figure 3). The results of ANOVA showed there was statistically difference in different levels of factor A (time) on MBP mRNA (*P* < 0.05), and no significant difference (*P*>0.05) found in factor B (dose) and factor C (time-dose interaction) (Table 6). LSD showed that a statistically significance (*P* < 0.05) between therapeutic time 1.0 h (A1) and 1.5 h (A2), 1.0 h (A1) and 2.0 h (A3), 1.5 h (A2) and 2.0 h (A3), 1.5 h (A2) and 2.5 h (A4), 2.0 h (A3) and 2.5 h (A4), but no statistical difference in the rest levels (*P* > 0.05) as well as the therapeutic dose levels (*P* > 0.05). Consideration of A2B3 combination is the best, so the best treatment time window and dose is 1.5 h, 20 mg/kg.

**Table 6:** ANOVA of RAM

**Source of variation**	**SS**	**Df**	**MS**	***F***	***P***
Time window	0.316	3	0.105	46.73	0.01
Drug dose	0.005	3	0.002	0.70	0.59
Time × dose	0.026	3	0.009	3.84	0.08
Error	0.014	6	0.002		

## Discussion

Orthogonal array is an efficient and economical test, which has the advantage of balancing samples and reducing the test times at the same time, so that each test has a strong representation [15]. In this study, we applied the orthogonal experiment to overall design, comprehensive comparison and statistical analyse, to find better treatment options through the small number of experiments to achieve the best therapeutic effect.

Myelin basic protein (MBP), including central and peripheral MBP, has the highest content in while matter. As a kind of basic membrane proteins with no sugar and lipid, MBP is synthesized by oligodendrocytes in central nervous system [16]. Only nerve fibers with myelination can complete its conduction function, so that MBP is an important structural protein involved in the synthesis of myelin and plays an important role in nervous system, such as insulation and fast conduction in nerve fiber [17,18]. The transcription products of MBP gene in human and mouse are different. There are at least 5 kinds of products including 21.5 kD, 18.5 kD, 17a, 17b and 14 kD in mouse brain. However, there are only 4 kinds of products consisting of 21.5 kD, 20.2 kD, 18.5 kD and 17.3 kD in human brain, of which the 18.5 kD MBP is the main protein of mature myelin in central nervous system [19,20]. Normally the concentration of MBP in cerebrospinal fluid (CSF) is lower than 6.95 mg/L. After cerebral ischemia, the ischemia and hypoxia of brain tissue can lead to oligodendrocyte death and demyelination, so caused MBP flowing into CSF. In addition, cerebral injury can damage the structure of blood–brain barrier (BBB) and cause MBP leaked in CSF passing through BBB into blood. Therefore, determining the serum level of MBP could partly reflect whether there was brain injury or not, and MBP level in serum also become a specific marker protein to judge demyelination [21-23]. Previous animal experiments showed that the expression of MBP mRNA and protein reduced in the early period of cerebral ischemic injury, especially in the first 24 h, and the content of MBP protein decreased significantly [24]. Recently, some experiments [4,25,26] reported that MBP played an important role in the prediction of the severity of brain injury and the prognosis, and the increased expression of MBP played a role in the protection of brain [27]. In this experiment, we determined the expression of MBP mRNA and protein to evaluate the degree of brain injury. The results showed that after cerebral ischemic injury, the expression of MBP mRNA and protein significantly decreased; and LFB staining and TEM showed that myelin fibers synchronous reduced and the damaged-positive cells were significantly increased, thus confirming that the detection of MBP at all levels can be a biological indicator of brain injury and demyelination.

Picroside II is an active ingredient of *Picrorhizae,* which pharmacological functions consist of cleaning heat, drying humidity, alleviating fever, eliminating dampness, retreating steam, cooling blood and cholagogue [28]. Li et al. [29] confirmed that picroside II had antioxidant effect and could reduce the H_2_O_2_-induced injury in PC12 cells to improve the cell survival [30]. Our research team found that picroside II could inhibit the expression of inflammatory factors such as Toll-like receptor 4 (TLR4), nuclear factor κB (NFκB), caspase enzymes-3 (caspase-3), and tumor necrosis factor α (TNFα) in cerebral ischemic penumbra after middle cerebral artery occlusion and reperfusion, and then inhibit neuronal apoptosis induced by ischemia [31-35]. This experiment results showed that comparing with the model group, the myelin nerve fibers arranged in order, vacuolar cells decreased, the expression of MBP and the transcription levels of MBP mRNA increased on different degrees after treatment by picroside II. These results proved the neuroprotective effect of picroside II against cerebral ischemic injury from various aspects and levels. Further time window and therapeutic dose optimization showed that injecting picroside II 10-20 mg/kg body weight intraperitoneally at ischemia 1.5 h-2.0 h could be achieved a significant effect against cerebral ischemic injury.

## Conclusion

Given the principle of lowest therapeutic dose with longest time window, the optimized therapeutic dose and time window should be injecting picroside II intraperitoneally with 10-20 mg/kg body weight at ischemia 1.5-2.0 h in cerebral ischemic injury in rats.

## Methods

### Establishment of animal models and grouping

Total of 200 adult healthy male *Wistar* rats (weighted 230-250 g, SPF grade) were supplied by the Experiment Animal Center of Qingdao Drug Inspection Institute (SCXK (LU) 20100100). This experiment was approved by the Ethics Committee of Qingdao University Medical College (QUMC 2011–09). The local legislation for ethics of experiment on animals and guidelines for the care and use of laboratory animals were followed in all animal procedures. All animals were acclimatized for 7d humidity-controlled housing with natural illumination and allowed to eat and drink freely at room temperature (23 ± 2°C). Fifteen (5 × 3) rats were randomly selected for control group, and the rest 185 rats were anesthetized by injecting intraperitoneally 10% chloral hydrate (3 ml/kg) after fasting for 12 h and fixed in supine position to conduct aseptic operation strictly. The bilateral common carotid arteries were colligated to establish forebrain ischemic models (BCCAO) [36,37]. The rats of control group (5 × 3) were operated as the same experimental procedures besides BCCAO. Core body temperature was keeping with a rectal probe and maintained at 36-37°C using a homeothermic blanket control unit during and after the surgery operation. Twenty-six rats un-awakened or died after 2 h of operation were rejected out, while 159 successful models whose cerebral blood flow curve (PeriFlux 5000, Sweden) dropped to 30% were brought into the experiment and were randomly divided into model group (5 × 3 cases) and treatment group (16 × 3 × 3 case).

### Orthogonal experimental design and intervention

The treatment group rats (16 × 3 × 3 case) were sub-grouped according to the principle of orthogonal experimental design of [*L*_16_(4^5^)] consisting of two impact factors with four impact levels. The impact factor A is the therapeutic time widow designed four levels as 1.0 h, 1.5 h, 2.0 h, 2.5 h after ischemia. The impact factor B is the therapeutic drug dose which has four levels as following 5 mg/kg, 10 mg/kg, 20 mg/kg and 40 mg/kg body weight. The orthogonal experimental test was repeated 3 times.

Picroside II (CAS No: 39012-20-9) which the purity exceed 98% and molecular formula is C_23_H_28_O_13_, supplied by Tianjin Kuiqing Medical Technology Co. Ltd. According to the weight of rats, corresponding dose of picroside II powder was taken and diluted into 1 ml solution by isotonic saline solution and injected intraperitoneally according to the corresponding designed doses at designed time in the orthogonal layout [*L*_16_(4^5^)]. Rats in control group and model group were intraperitoneally injected the same dose saline after cerebral ischemia 2 h. The brain tissue was took out to evaluate the therapeutic effect of picroside II after treatment 24 h.

### Fast green staining(FGS)

The rats from control group (5 cases), model group (5 cases) and treatment group (16 × 3 cases) were randomly chosen and anesthetized by injecting intraperitoneally 10% chloral hydrate (3 ml/kg), and perfused by normal saline 200 ml and fixed by 4% paraformaldehyde solution 200 ml successively via heart. Then the whole brain was taken out and post-fixed in 4% formaldehyde solution for 2 h and soaked in distilled water for 4 h. After conventional gradient ethanol dehydration, xylene transparent, paraffin embedding, coronal sections with a thickness of 5 μm were continuously cut forward from the posterior of optic chiasma by a microtome (Leica CM2027, Germany) and then adhered on the slices processed with poly-lysine.

The paraffin sections were dewaxed by dimethyl benzene and washed routinely, dyed 1 h in fast green alcohol solution at 37°C after dealing with 95% alcohol 1 min, and then washed by 95% alcohol (10 s × 2 times) and distilled water (15 s × 3 times), putted in 0.3% lithium carbonate to separate color, re-stained 90s by nuclear fast red after washing by distilled water (15 s × 3 times), finally washed by distilled water and conventionally dehydrated by gradient ethanol, cleared by xylene and sealed with neutral balsam. Normal myelin showed cord-like, dark green, tightly packed, and cells were red under light microscope. Five non-overlap visual fields at ischemic area were randomly observed under 400-fold light microscope. Quantity One software was used to analyse the gray value and took the mean. The change of myelin was presented by myelin gray value (MGV = MBP gray value – background gray value).

### Transmission electron microscopy (TEM)

Ultrathin sections: Took some fresh brain tissue from the ischemic area and cut into pieces of 1 mm × 1 mm × 1 mm, fixed with 2.5% glutaraldehyde for 24 h and 1% osmium tetroxide for 2 h and dehydrated by graded series of acetone. Then soaked with the mixture of acetone and embedding solution (1:1) for 1.5 h and pure embedding solution overnight at 37°C respectively. The samples were put into the embedding plate filled with epoxy resin Epon812 to form embedding blocks eventually. The 50 nm ultrathin sections were cut by the ultramicrotome (Leica EM UC6, Germany) and placed on the nets prepared with polyvinyl formal, stored at 4°C.

TEM: Dripped a drop of 3% uranyl acetate-alcohol saturated solution (pH = 3.5) in a petri dish, covered the nets of ultrathin sections to contact with the dye liquor to stain for 30 min, and rinsed with double-distilled water for 10 min × 3 times to suck up water. Then, covered the nets of ultrathin sections to a drop of 6% lead citrate dye liquor (pH = 12) in another petri dish to stain for 5 min, rinsed with non-carbon dioxide double-distilled water for 10 min × 3 times, dried at room temperature. The ultrastructure of myelin was observed under TEM (JEM-1200EX, Japan).

### Immunohistochemical (IHC) assay

Paraffin sections prepared as above were dewaxed and washed routinely, operated by the specification of SABC kit, developed by DAB chromogenic reagent kit and re-stained by hematoxylin (all kits were provided by Wuhan Boster Biotech Co. Ltd). Under light microscope myelin showed claybank streak and positive cells’ cytoplasm presented uneven coloring, vacuolization in cells. Negative control slices were dyed with 0.01 mol/L PBS instead of rabbit anti-rat MBP primary antibody and no positive reaction appeared. Five non-overlap visual fields randomly at ischemic area were chose under 400-fold light microscope. The gray value was analysed by the software of Quantity One and last took the mean. The expressing intensity of MBP was presented by gray value of myelin (GVM = MBP gray value – background gray value).

### Western blot (WB)

Extraction of total protein: After treatment 24 h, we randomly chose 5 rats from control group, 5 from model group and (16 × 3) from treatment group to perfuse from heart with normal saline 200 ml following anesthetizing by 10% chloral hydrate. Took 200 mg ischemic brain tissue and put it into 1.5 ml EP tubes, added cell lysis buffer as the proportion of 1:4 (No. P0013, Biyuntian Biotech Co. Ltd., China), then grinded fully and homogenized by ultrasonic wave at −4°C ice bath, and collected the supernatant in another EP tube after centrifuging with 10,949 g for 10 min at 4°C (Eppendorf 5801, Germany). The BCA-100 protein quantitative kit (Shenneng Biotech. Co. Ltd., China) was used to determine the protein content, then stored at −20°C.

Western blot: MBP proteins (18, 23 kD) were separated by sodium dodecyl sulfate polyacrylamide gel electrophoresis (12% separating gel on 75 V and 5% concentrated gel on 120 V successively) and transferred onto a polyvinylidene difluoride membranes (40 min with 360 mA). Phosphate buffered saline with Tween-10 (PBST) was used to wash the gel films 5 min by 3 times, then the films were added rabbit anti-rat MBP primary antibodies (1:450, Abcam Company, Ab40390) to incubate 2 h and washed by PBST for 10 min by 3 times, then incubated 1 h in horseradish peroxidase goat anti-rabbit antibodies (1:10000, Beijing Golden Bridge Biotech. Co. Ltd., ZB-2301), finally washed with PBST and PBS successively for 5 min by 3 times. The gel film images was developed in A-B mixed developing agent and scanned with Bio-Rad-2000 gel-imaging system to analysed gray value of strap by Quantity One software. In the same specimen, the gray value of β-action (42 kD), as an internal parameter, was also detected to calibrate the content of each target protein. The relative content of protein (RCP) = the gray value of MBP/the gray value β-action. The experiment was repeated 3 times and the results presented with mean ± standard deviation.

### Reverse transcription polymerase chain reaction (RT-PCR)

Extraction of total RNA: Chose 5 rats from control group and model group respectively and (16 × 3) rats in treatment group randomly and anesthetized by chloral hydrate after treatment 24 h. Took 200 mg ischemic brain tissue and put into 1.5 ml EP tube. Added RNA-Solv reagent 1 ml, minced and grinded, oscillated ultrasonically for 30 s and placed 5 min at room temperature, and centrifuged (4°C 12,000 g) for 15 min. Took the supernatant into another EP tube and added chloroform 0.2 ml, shocked and mixed 15 s, placed on ice for 10 min and centrifuged (4°C 12,000 g) for 15 min. Then, collected supernatant into another EP tube and join isopropyl alcohol 0.5 ml, blended gently, then placed on the ice for 10 min, centrifuged (4°C 12,000 g) for 15 min and discarded supernatant. Washed precipitation using 1 ml 75% alcohol, mixed and centrifuged (4°C 7,500 g) for 5 min, then abandoned supernatant carefully, dried 30 min (precipitation changed from white to transparent) in fume hood, and put in 57°C water bath for 10 min after adding 0.1% DEPC-H_2_O 30 μl. The purity and abundance of RNA were determined by ultraviolet spectrophotometer (Bekamann DU640, USA) and stored at −20°C.

RT-PCR: (1) Primers were designed with Premier 5.0 software and synthesized by Shanghai Invitrogen Co. Ltd. Target gene NSE (103 bp), sense primer: 5'–CCC ATT GGT GCA CAC TAA CCT-3', antisense: 5'-CGA CTT GAT TCA GCG ACA GGA-3'; GAPDH (110 bp) as an internal parameter, sense primer: 5'-CGT TGA CAT CCG TAA AGA CCT C-3', antisense: 5'-TAG GAG CCA GGG CAG TAA TCT-3'. (2) Reverse transcription synthesis system of cDNA (25 μl in total): Oligod T 2 μl and RNA 2 μg with DEPC-H_2_O added to 13.4 μl, and the mixed liquid was placed at 70°C for 5 min, then ice-bath for 5 min. Plused M-MLV RT 5 × 5 μl, dNTP mixture 5 μl, RNase inhibitor 0.62 μl and M-MLV RTase 1 μl. Then the mixed liquid was placed at 42°C for 1 h, reacted at 70°C for 15 min, and finally preserved at −20°C. (3) PCR system (50 μl): 5 μl 10 × PCR buffer, 1 μl dNTP (10 mmol/L), 1 μl cDNA, 1 μl primer1 (10 um), 1 μl primer2 (10 um), 0.4 μl Taq polymerase, with 0.1% DEPC-H_2_O added to 50 μl. PCR condition: The cDNA of MBP and GAPDH were amplified for 30 cycles, at 95°C for 3 min, 94°C for 30 s, 58°C for 30 s, and 72°C for 40 s, and finally extension at 72°C for 3 min. (4) Electrophoresis: 50 μl RT-PCR system with 10 μl 6 × DNA loading buffer added into, was shocked and blended, and then centrifuged for 5 s, followed with 10 μl sample loaded. Appropriate DNA Marker was selected to load 2 μl, and followed with 2% agarose gel electrophoresis (120 V/100 mA) for 30 min and ethidium bromide (EB) staining. Quantity One software was used to analyse the gray value after scanning by Bio-Rad-2000 gel-imaging system. The results were presented as relative abundance of mRNA (RAM): the gray values of MBP mRNA/GAPDH mRNA. The results were repeated 3 times and expressed with mean ± standard deviation.

### Statistical analysis

Determination of statistical significance was carried out with Student’s *t*-test between two groups. One-way analysis of variance (One-way ANOVA) was used for the comparison of multiple sets of data, then further study was made by Least significant differences (LSD) to compare multiple data. All datum statistically analysed by SPSS 17.0 software. Values were considered to be significant when *P* was less than 0.05.

## Competing interests

The authors declare that they have no competing interests.

## Authors’ contributions

LZ and YG were designed the study protocol. LZ, XJ and MZ collected the data. LZ analyzed and interpreted the findings and wrote the manuscript. YG performed a critical revision of the manuscript for important intellectual content and was responsible for submitting the final manuscript. All authors read and approved the final manuscript.
